# Review and reflections about pulsatile ventricular assist devices from history to future: concerning safety and low haemolysis—still needed

**DOI:** 10.1007/s10047-020-01170-3

**Published:** 2020-05-04

**Authors:** Inge Köhne

**Affiliations:** Department for Health Services Research, Carl von Ossietzky University of Oldenburg, Ammerländer Heerstr. 114-118, 26129 Oldenburg, Germany

**Keywords:** Pulsatile ventricular assist device, Extracorporeal circulation, Pediatric ventricular support, Complications with ventricular assist devices, History of heart assist

## Abstract

Since the first use of a ventricular assist device in 1963 many extracorporeal and implantable pulsatile blood pumps have been developed. After the invention of continuous flow blood pumps the implantable pulsatile pumps are not available anymore. The new rotary pumps spend a better quality of life because many of the patients can go home. Nevertheless, the extracorporeal pulsatile pumps have some advantages. They are low-cost systems, produce less haemolysis and heart-recovery can be tested easily. Pump failure is easy to realize because the pumps can be observed visually. Pump exchange can be done easily without any chirurgic surgery. As volume displacement pumps they can produce high blood pressure, so they are the only ones suitable for pediatric patients. Therefore, they are indispensable for clinical use today and in the future. In this work, nearly all pulsatile blood pumps used in clinical life are described.

## Introduction

Total Artificial Hearts (TAH) only represent a minority of blood pumps used to date, the vast majority are used parallel to the heart as a Ventricular Assist Device (VAD). Many VADs that have been developed help support the diseased heart work to provide adequate blood flow. Some hearts were able to recover to the point where a transplant was no longer necessary, and the patients were considered cured [1]. Since the number of available donor hearts worldwide does not meet the demand, heart disease patients are given different VADs until transplantation time, so they can have a chance to survive the waiting period for a donor’s heart. It is believed that there will be more and more patients urgently waiting for a donor’s heart. In addition, VAD is now used in patients who are not eligible for a heart transplant, as destination therapy. This work describes all pulsatile VAD systems (P-VAD), which were used in patients and gives an overview of complications, risks and outcome.

## Not implantable pulsatile VAD

**Fig. 1:**
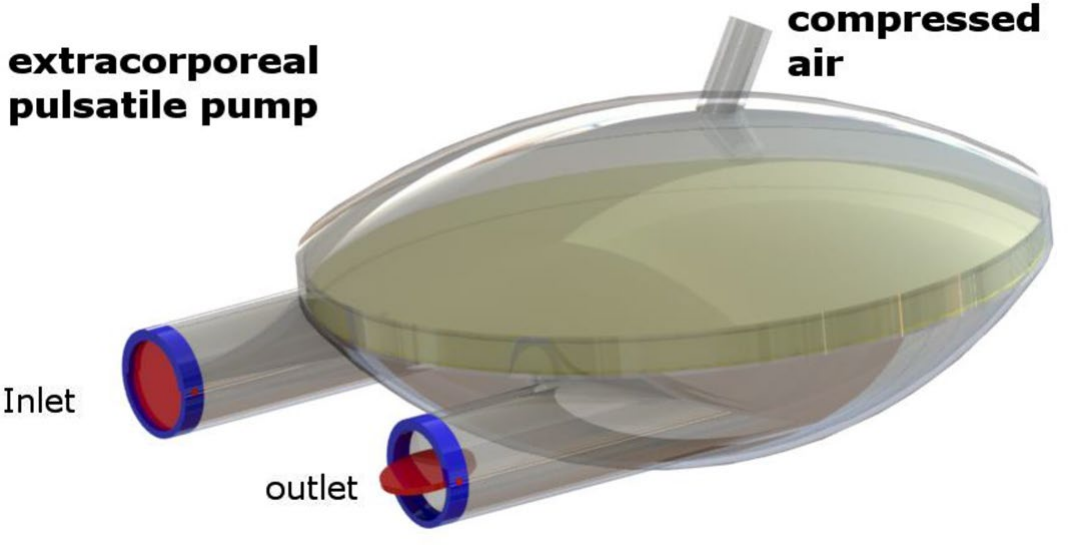
Principle model of an extracorporeal pneumatically driven pulsatile blood-pump

In the early days of artificial heart research, it was assumed that the blood, like with the natural heart, had to be transported through the body in a pulsatile manner. Therefore, pulsatile volume displacement pumps have been developed for cardiac support. These are today referred to as the first generation of blood pumps [2]. The first VADs were usually extracorporeal pumps used outside the body. They consist of a plastic housing, which is divided by a movable membrane into two chambers. The blood chamber is equipped with a separate inflow and outflow, wherein the desired flow direction is controlled by artificial heart valves. The air chamber is connected to a drive system that injects and releases compressed air. By filling the air chamber, the membrane is moved, and the blood is forced out of the blood chamber (Fig. 1). The blood pump is connected to the heart and aorta or pulmonary artery via two cannulas through the skin. These extracorporeal pumps can be used as a Left Ventricular Assist Device (LVAD) or as a Right Ventricular Assist Device (RVAD). Both pumps can also work together as bilateral support (bi-VAD). They are available in many sizes, so they are used all over the world, from infants to adults. All extracorporeal P-VADs used in patients to date are described below. A comparison with technical data can be found in Table 1.

**Table 1:** Overview of pulsatile extracorporeal VAD used in human patients

Name	Manufacturer	Drive	Performance	Dimensions	First use	No. of patients
DeBakey Pump	Texas Heart Institute Houston, USA	Pneumatic CO_2_	3.5 l/min 80 mmHg		1966	3
Ellipsoid VAD	Medizinische Universität Vienna, Austria	Pneumatic air			1977	11
New Vienna Pulsatile VAD	Medizinische Universität Vienna, Austria	Pneumatic air			1986	9
Thoratec PVAD	Thoratec Corp Pleasanton, USA	Pneumatic air	7 l/min	65 ml	1981	> 5000 Still in use
EXCOR	Berlin heart Berlin, Germany	Pneumatic air	12 l/min 350 mmHg	50/60/80 ml	1985	> 2200 Still in use
EXCOR Pediatric	Berlin heart Berlin, Germany	Pneumatic air	4.5 l/min 350 mmHg	10/15/25/30 ml	1992	> 2000 Still in use
MEDOS HIA	Medos medizintechnik Stolberg, Germany	Pneumatic air		10/25/60/80 ml	1994	> 650
Abiomed AB5000	Abiomed Inc Danvers, USA	Pneumatic air	4.5 l/min	80 ml		
Abiomed BVS5000	Abiomed Inc Danvers, USA	Pneumatic air	6 l/min	82 ml	1984	> 6000 Max. 14 days
POLVAD	Plastmed Ltd Zywiec, Poland	Pneumatic air		80 ml	1993	Still in use
ZEON VAD	ZEON Co. Ltd Tokyo, Japan	Pneumatic air	7.5 l/min	40/50/60 ml	1980	160
Toyobo/Nipro VAD	Nipro Corp Osaka, Japan	Pneumatic air	7 l/min	20/60 ml	1982	> 900 Still in use
Lou-Ye pump	Cardiovascular Institute Guangdong, China	Pneumatic air	4.8 l/min	80 ml	1998	18

### DeBakey Left Ventricular Bypass Pump, Texas Heart Institute, Houston, USA

After the first pneumatically driven VAD was implanted in 1963, DeBakey and Liotta developed a similar, non-implantable VAD. In 1966 it was used in three patients. The ventricle was made of silicone, coated inside with a fabric made of Dacron fibre. The casing of the pump was spherical and divided by an elastic membrane into blood and a gas chamber. CO_2_ was used as a propellant. Two ball valves were used as heart valves. It was possible to regulate the frequency of the pump via the patient’s ECG. The blood pump delivered a maximum of 3.5 l/min at a blood pressure of 80 mmHg. The longest duration of use was 10 days. All patients recovered and could be discharged [3–5].

### Ellipsoid LVAD und New Vienna Pulsatile VAD, Medizinische Universität Wien, Austria

The Medical University in Vienna has been working on the development of VAD and TAH since 1970. The ellipsoid VAD was developed together with the ellipsoid TAH under the direction of Unger. The first use of the Ellipsoid VAD took place in Vienna in 1977, a total of 11 patients were supported [6, 7]. From 1985 on the New Vienna Pulsatile VAD was developed together with the New Vienna TAH in the newly formed Artificial Heart Research Group. It consisted of a vacuum-formed pellethane ventricle. The pumping capacity was 3.5–4.5 l/min. Despite initial success in nine patients, no industrial company could be found to manufacture the VAD or TAH [7, 8].

### Thoratec PVAD, Thoratec Corporation, Pleasanton, USA

2015 acquired by St. Jude Medical, since 2017 Abbott Laboratories, Pleasanton, USA. The Thoratec Paracorporeal Ventricular Assist Device evolved from the Pierce-Donachy VAD. First clinical trials began in 1976, and since 1981 the VAD has been used to treat cardiogenic shock following cardiac surgery. It was used for the first time in 1984 to bridge the gap until a heart transplant was performed. Thoratec PVAD received FDA approval in 1995 and CE approval in 2003. The ventricle is seamlessly manufactured from polyurethane with a two-layer membrane. The outer membrane being the support layer with its appropriate strength and elasticity, and the inner layer having a special lining to protect against thrombosis. The ventricle is located in a solid polysulfone housing. The cannulas are also made of polyurethane. The pump volume of the artificial ventricle is 65 ml. The maximum pump rate is 7 l/min. The Thoratec PVAD can be used as LVAD, RVAD, or bi-VAD. Until 2019 more than 5000 patients were treated with the Thoratec PVAD. It is one of the few extracorporeal pulsatile pumps still in use today [9–11].

### MEDOS HIA, Medos Medizintechnik GmbH, Stolberg, Germany

since 2017 part of Xenios AG, subsidiary of the Else Kroener-Fresenius Stiftung. The MEDOS HIA was developed at the Rheinisch-Westfälische Technische Hochschule in Aachen in 1982. Medos Medizintechnik GmbH was founded in 1987 to produce the system. The LVAD blood pumps were available in sizes 10, 25, 60 and 80 ml, however, with the bi-VAD the stroke volume for the right-hand pump was 10% lower. The pump was made of polyurethane with a double layer membrane. They used self-developed three-wing polyurethane valves as heart valves. It was first used on patients in 1994. In 1997, the system received CE approval for use in adults, children and infants. Until production was discontinued in 2011, MEDOS HIA was used in over 650 patients [12, 13].

### Berlin Heart, today EXCOR^®^, Deutsches Herzzentrum Berlin, Germany

then Mediport Kardiotechnik GmbH, renamed in Berlin Heart GmbH, Berlin, since 2006 subsidiary of the Syscore GmbH, Germany. The extracorporeal Berlin Heart VAD has been developed since the early 1970s under the direction of Bücherl at the Freie Universität Berlin. The first prototype, the Bücherl VAD, was used on a patient after heart surgery in 1985. In 1987, the research program for mechanical circulatory support came to the German Heart Institute in the Rudolf Virchow Hospital. The VAD was first used there in 1988 as a bridge for transplantation. Since 1992, the Berlin Heart has also been produced in smaller sizes for small children and infants. Since Berlin Heart launched the implantable axial blood pump INCOR in 2002, the extracorporeal pump has been called EXCOR, the pumps for infants and children EXCOR Pediatric. The EXCOR consists of one or two blood pumps, the pneumatic drive system and the connection cannulae. The blood pumps are available in the sizes 80, 60, 50, 30, 25, 15, and 10 ml stroke volume. The housing is made of polyurethane and is divided into a blood chamber and an air chamber by a three-layer membrane (graphite powder in between). Two of the three membranes are driving membranes, which absorb the mechanical load. The thinner blood-side membrane is designed that it is only moved by the other membranes and is not exposed to any stress. Since 1994, the blood chamber has been heparinized according to the Carmeda method to minimize thrombus formation [14, 15]. The large blood pumps (50–80 ml) are available both with two-disc metal heart valves and with three-bladed polyurethane heart valves, which are developed and manufactured by Berlin Heart itself. The small blood pumps always have three-winged heart valves. Customized silicone cannulas are connected to the heart valves and coated with Dacron fabric on the outside which guarantees protection against infections. Berlin Heart has developed the IKUS 2000 drive system especially for the small blood pumps, which can guarantee the high pressures of 350 mmHg (systole) and − 100 mmHg (diastole) required for these pumps. As of 2002, the mobile unit EXCOR-Driver is also available with two identical drive systems for redundancy. With the integrated rechargeable batteries, independent operation of up to 6 hours is possible. The EXCOR driver is so small and light that it can be carried comfortably by the patient on a trolley or as a backpack [16–18]. In Europe, the Berlin Heart EXCOR received CE approval in 1996. By 2015, the system had already been used on more than 2200 patients [19]. The EXCOR Pediatric is also approved in the USA (FDA approval 2011) [20]. In Japan, the first EXCOR Pediatric 2012 was used on a 14-month-old infant, and the system has been approved since 2015 [21]. Until 2012, the longest period of use was 877 days for a 6-year-old child [22]. At the beginning of 2018, EXCOR was approved in 53 countries and EXCOR Pediatric in 41. More than 2000 children have been treated with the EXCOR Pediatric to date [23]. Even in times when the VAD is continuously working, EXCOR is still used, mainly for bilateral cardiac support and in children. The system is inexpensive, making it very popular in countries with limited financial resources [19].

### Abiomed AB5000, ABIOMED Inc., Danvers, USA

The structure and functionality of the Abiomed AB5000 were similar to those of Thoratec and Berlin Heart. The AB5000 received FDA approval in 2003 and CE approval in 2008. The blood chamber and the heart valves were made of polyurethane. A special feature of the Abiomed AB5000 was that the outlet was located in the middle of the blood chamber, which was supposed to have a positive effect on the blood flow. The system achieved a pumping capacity of 4.5 l/min. The blood pump is no longer being produced [24, 25].

### Abiomed BVS 5000, ABIOMED Inc., Danvers, USA

The Abiomed BVS500 system was available for short-term cardiac support up to 14 days. It has been used in clinical trials since 1984 and has been approved by the FDA for short-term heart support since 1995, as well as in Europe. The system consists of two tubular polyurethane blood chambers connected in series, separated from each other by three-pronged angioflex heart valves. The blood chambers are located in a rigid plastic housing and can be compressed separately pneumatically. The first chamber functions as an atrium into which the blood can flow continuously, only triggered by gravity, a vacuum to aspirate the blood are not necessary. If the atrial chamber is full, the blood is pumped into the next main chamber. From the main chamber it is then pumped into the body at high pressure. The pump creates a flow of 6 l/min, with a ventricle filling of 80 ml. The system was quite inexpensive at $15,000, plus the cost of the drive and control console. The BVS 5000 was used more than 6000 times but is no longer being manufactured [26, 27].

### POLVAD-II, Plastmed Ltd., Zywiec, Poland

The POLVAD was developed at the Silesian Medical Academy in the Artificial Heart Laboratory in Zabrze. The first clinical use of the POLVAD took place in 1993. The design of the POLVAD essentially corresponds to that of the Berlin Heart EXCOR with a polyurethane housing, a three-layer membrane with graphite lubrication and Sorin heart valves. The POLVAD has a heartbeat volume of 80 ml and is driven by a JSN-201 driving unit (OBREAM, Zabrze) [28, 29].

### ZEON VAD, ZEON Co. Ltd., Tokyo, Japan

The ZEON VAD was developed at Tokyo University and first used in a patient in 1980. It marked the start of the use of VAD in Japan. Although heart transplants were not allowed in Japan at the time, these pumps were used there earlier than in the USA and Europe. The ZEON VAD were bag pumps with a pump volume of 40, 50 or 60 ml. They achieved a pumping capacity of 7.5 l/min with a rate of 80–100 bpm (beats per minute). The blood chamber is completely flexible and installed in a fixed housing. By blowing air into the housing, the blood bag is compressed from all sides and the blood is pumped into the body. Blood bag and cannulas were made of PVC, all surfaces were coated with Cardiothane. Björk-Shiley valves were used. The ZEON VAD was used in 160 patients until sales were discontinued in 2005 [30, 31].

### Toyobo VAD, Toyobo, later NIPRO-VAD, Nipro Corporation, Osaka, Japan

The Toyobo VAD, today NIPRO-LVAD, has been used successfully in Japan since 1982, and by 2012 it had been used in 902 patients. The blood pump consists of polyether and polyurethane, with Medtronic Hall heart valves. The pump can pump 50–60 ml of blood per “heartbeat”. In addition, there is a pump for children with a stroke volume of 20 ml. The longest continuous use was 1264 days, with a survival rate of 84% after 1 year [31, 32]. Besides the ZEON VAD and the Toyobo VAD, the bag pumps of Tohoku University were tested in three patients and the Thomas Giken diaphragm pump in 14 patients in Japan [33].

### Lou-Ye Pump, Guangdong Provincial Cardiovascular Institute, China

The Lou-Ye Pump was developed in 1990 and was first used in patients in 1998. The pump has a stroke volume of 80 ml and a fixed stroke rate of 60 bpm. To facilitate the emptying of the blood chamber, the inlet and outlet are arranged crosswise and are not parallel to each other, as is the case with all other diaphragm pumps. Up to 2014, this pump had been used in a total of 18 patients. A small pump for children with a stroke volume of 20 ml is under development. Attempts to coat the inside of the blood chamber with endothelial cells to reduce the risk of thrombosis have not been successful so far [34, 35].

## Implantable pulsatile VAD

To increase patient comfort, implantable VADs were designed parallel to the extracorporeal blood pumps. These intracorporeal VADs can be divided into pulsatile and continuous pumps. Similar to extracorporeal pumps, the pulsatile pumps operate with a movable diaphragm. This membrane can be moved either by compressed air or by an electrically driven pressure plate. Here, too, the blood flow is controlled by valves. Since the pumps are implanted, only the drive energy supply has to be led through the skin to the outside. These pumps were very large, so they were only used as LVAD in adult patients. With the development of the much smaller continuous flow blood pumps, the number of implanted pulsatile VADs has greatly decreased. None of the systems have been available since 2019. The pulsatile implantable VADs previously used in patients are described below. A comparison with technical data in Table 2.

**Table 2:** Overview of pulsatile implantable VAD used in human patients

Name	Manufacturer	Drive	Performance	Dimensions	First use	No. of patients
DeBakey Pump	Texas Heart Institute Houston, USA	Pneumatic CO_2_	2.5 l/min		1963	1
TECO Model-7 ALVAD	ThermoElectron Waltham, USA	Pneumatic air	10 l/min	*D* = 6, 3 cm; *l* = 17 cm 300 cm^3^, 470 g	1975	23
Novacor N100	Baxter Health Care Oakland, USA	Electric Linear motor	12 l/min	16 × 13 x 6 cm 1 kg	1984	> 1800
HeartMate IP-LVAS	TCI Woburn, USA	Pneumatic air	12 l/min	83 ml; 570 g	1986	1278
HeartMate VE/ XVE-LVAS	TCI Woburn, USA	Electric	10 1/min	1150 g	1991	> 4100
Thoratec IVAD	Thoratec Corp Pleasanton, USA	Pneumatic air	7.2 l/min	252 cm^3^; 339 g	2001	> 560
LionHeart LVAD	Arrow International Reading, USA	Electric	8 l/min	700 g + 1500 g	1999	23

### DeBakey/ Liotta Left Ventricular Bypass Pump, Texas Heart Institute, Houston, USA

The first VAD, developed by DeBakey and Liotta, was used as a pumping aid after heart surgery in 1963. However, thrombi formed after 48 h, so the heart support had to be stopped after 4 days. It was a tubular pump made of silicone reinforced with Dacron fibre that pumped the blood from the left atrium directly into the aorta. The peristaltic pump was divided lengthwise into a blood chamber and a gas chamber by a partition wall with ball valves as heart valves. The pumping effect was created by pumping CO_2_ in and out, which compressed the blood chamber and pulled it apart again. The pump generated a volume flow of 1.8–2.5 l/min [4, 36].

### TECO Model-VII ALVAD, Texas Heart Institute, Houston and ThermoElectron, Waltham, USA

In 1968, the TECO Model VII ALVAD (abdominal left ventricular assist device) was developed from the prototype of DeBakey and Liotta at the Texas Heart Institute together with the company ThermoElectron and with the support of the NHLI (National Heart and Lung Institute). It was the second implantable VAD to be inserted into a human. The centrically located blood chamber consisted of a silicone tube, later polyurethane, which was built into an ellipsoidal housing of stainless steel, later titanium, with two single-disc heart valves. The blood chamber was lined on the inside with polyester fibre to allow blood cells to accumulate. The drive was pneumatic. The system could pump 10 l/min blood at 140 bpm. It was first used in a human being in 1975 and used a total of 23 times by 1980. In 1978, the blood pump was inserted into a 21-year-old patient, who survived for 5 days until a donor heart was implanted. This was the world’s first use of a VAD as a bridge to transplantation [37, 38].

### Novacor N100, Novacor Medical Corporation, Berkley, USA

1988 acquired by Baxter Health Care Corporation, Oakland. 2000 sold to WorldHeart Corporation, Oakland. 2012 acquired by Heart-Ware International Inc. Framingham, USA. The Novacor N100 VAD has been developed in California since 1970. It was the first electrically powered implantable VAD when it was used in 1984 in a patient who was the first to live for a long time after a heart transplant. In 1990, the first patient survived more than 1 year with the system. From 1993 onwards, patients could be discharged home with the portable supply and control module. Novacor received CE approval in 1994, FDA approval in 1998 and approvals in Canada and Japan in 1999. A mark of 1000 supported patients was reached in 1999, and the first patient lived over 4 years with the system. The Novacor N100 is completely symmetrical. The blood chamber consists of a seamless circular soft polyurethane sack that is compressed from both sides by a pressure plate. It is driven by a linear motor and reset by leaf springs. Due to the symmetrical drive, only internal forces are generated; no forces or torques are transmitted to the surrounding epoxy resin housing. The power supply is provided by a cable with a silicone sheath, which is led through the skin together with the hose for air compensation. Heart valves made of denatured bovine or porcine pericardium with polyester cannulas are used. The control unit is compact and lightweight (0.65 kg) so that it can be carried by the patient on the belt, together with two rechargeable battery packs (1.6 kg) that can operate the pump for 6 h. A larger mobile console is available for stationary use. The volume of the blood chamber is 70 ml. The pump operates at a frequency of up to 180 bpm, which corresponds to a pump capacity of 12 l/min. The implantable part of the VAD measures 16 × 13 × 6 cm and weighs 1.0 kg. The system was withdrawn from the market in 2008 and the development of the successor Novacor two was also discontinued. A total of over 1800 Novacor N100 VADs were implanted [39, 40].

### HeartMate IP-LVAS, Thermo Cardiosystems Inc. (TCI), Woburn, USA

since 2000 Thoratec Corporation, 2015 acquired by St. Jude Medical, since 2017 Abbott Laboratories, Pleasanton, USA. The HeartMate IP-LVAS (Implantable Pneumatic Left Ventricular Assist System) was developed in 1975 by TCI in collaboration with the Texas Heart Institute. The first clinical use was in 1986. In 1994 it was approved by the FDA as the first system for bridge to transplantation. The disc-shaped pump housing is made of titanium, with a blood chamber made of polyurethane. The drive is pneumatic, the blood chamber is separated from the compressed air chamber by a membrane made of polyurethane. The blood chamber is coated on the inside so that blood cells can accumulate. Modified porcine aortic valves are used as heart valves. With a maximum stroke volume of 83 ml, the HeartMate IP can generate a blood flow of 11.6 l/min at 140 bpm. The blood pump weighs 570 g. The drive hose passes through the skin and is connected to a drive console. The console weighed 10 kg and could work 6–8 h independently with a battery. This allowed the patient great freedom of movement so that he could usually be discharged home. By 2002 it had been implanted in 1278 patients, with the longest duration being 751 days. Production has since been discontinued [41, 42].

### HeartMate VE-LVAS/XVE-LVAS, Thermo Cardiosystems Inc. (TCI), Woburn, USA

The HeartMate Vented Electric Left Ventricular Assist System essentially corresponds to the HeartMate IP-LVAS, it is equipped with an integrated electric motor drive. The 12-V DC motor moves a pressure plate that compresses the membrane of the blood chamber. One revolution of the motor causes one pumping cycle. The drive cable is led through the skin together with a hose for air exchange. The control unit with the battery is so small that it can be carried on a belt and lasts for 4–6 h. The HeartMate VE-LVAS was first used at the Texas Heart Institute in 1991 and received FDA approval in 1998. The performance data corresponds to that of the HeartMate IP LVAS. The enhanced version HeartMate XVE-LVAS received FDA approval in 2001, while the optimized version HeartMate SNAP-VE was the first VAD to receive FDA approval for destination therapy. The HeartMate XVE-LVAS received this approval in 2003. The system received CE approval in 2003. By 2018, 4100 HeartMate LVAS had been implanted worldwide and the longest period of use was 1146 days. Production of the electrical HeartMate variants has now been discontinued [11, 41–44].

### Thoratec IVAD, Thoratec Corporation, Pleasanton, USA

2015 acquired by St. Jude Medical, since 2017 Abbott Laboratories, Pleasanton, USA. The Thoratec implantable VAD was developed from the Thoratec PVAD. Its structure and performance correspond to the latter system, except that the housing is made of titanium. The blood pumps can thus be implanted. To monitor the pump work, an infrared sensor for volume measurement was integrated. The first use took place in 2001, and FDA approval was obtained in 2004. The pump weighs 339 g and requires a volume of 252 ml in the body. This means that two implanted pumps can be used for bilateral cardiac support in patients of the corresponding size. The maximum pumping capacity is 7.2 l/min. Until recently, it was the only implantable pulsatile heart support system on the market, but production was discontinued in 2019 [45, 46].

### LionHeart LVD-2000, Arrow International Inc., Reading, USA

The LionHeart LVD-2000 was developed by Pennsylvania State University, Hershey, in collaboration with Arrow International. The elastic blood chamber is housed in a titanium housing with two single-disc heart valves and an electric DC motor that moves a pressure plate. Since the pressure plate is not connected to the blood chamber, the filling is passive, without suction. In addition, a compliance chamber is implanted to ensure gas volume equalization in the pump housing. The third implanted part is the control unit in a titanium housing containing rechargeable batteries. The energy is supplied electromagnetically via coils through the skin. One coil is located under the skin, the energy is transmitted through another coil on the skin, whereby the internal batteries are also charged so that the pump can work briefly for up to 20 min without external energy supply. The blood pump weighs 700 g and the “accessory” to be implanted 1500 g. The pump has a stroke volume of 64 ml and can generate a volume flow of 8 l/min. The LionHeart was first implanted in a patient in Bad Oeynhausen in 1999. The system received CE approval in 2003. The longest period of use in the USA was 13 months. The LionHeart LVD-2000 was discontinued in 2005 [47, 48].

## Discussion

In contrast to other artificial implants, which can be replaced in case of failure, the failure of a VAD will very quickly lead to the death of the patient. Although all possible safety measures are adhered to, complications occur again and again. These are not only caused by the blood pumps themselves but can also be caused by previous damage to the patient’s body, the obligatory operation, the management of medication or infections.

### Pump failure

Since continuous flow pumps have been used as VADs (CF-VAD), the number of failed pumps has decreased considerably. While the pulsatile HeartMate XVE had 0.51 failures per patient and year, the axial pump HeartMate II had only 0.06 failures per patient and year [49]. The database of all VAD implants with FDA approval (Interagency Registry for Mechanically Assisted Circulatory Support—INTERMACS) carried out in the USA between 2006 and 2011 also shows a higher failure rate of pulsatile versus continuous flow pumps [50]. The main reason for failure is damage to the cable. Other causes of failure are rotor blockage due to thrombus formation or cannula detachment, either from the patient’s pump or tissue. While a failure of a pulsatile pump is not immediately life-threatening because the patient’s own heart can still provide some pumping power, a failure of a continuous flow pump causes the blood pumped from the natural heart into the aorta to flow through the pump back into the ventricle because the pump has no valves. This leads to increased pressure and blood volume in the ventricle and a decrease in blood flow to all body organs [51]. A comparison between the HeartMate II axial pump and the HeartWare HVAD radial pump between 2004 and 2016 showed that pump failure was only directly caused by pump damage in 13% of cases. The most common cause of failure was a control system failure, followed by battery failure and cable system failure [52]. A phenomenon that has rarely been investigated so far is a pump failure due to wrong doing of the patient. Mistakes or forgotten information on the part of the patient, family members and nursing staff often leads to complications. The patient or caregiver can drop the control unit, insert batteries incorrectly, or connect cables incorrectly. It is possible to forget to reconnect the control console after a battery change. Often, patients do not know how to respond properly to error or alarm messages from their control unit [53].

### Bleeding, von-Willebrand-Syndrome and Haemolysis

There is no universal definition of when bleeding is considered severe when using VAD [54]. The most common type is gastrointestinal bleeding. These occur more frequently when VAD does not produce a pulsatile blood flow. If a heart transplant is performed after cardiac support, these bleedings usually stop [55]. The number of patients who have received a successful heart transplant after heart support is lower if there is heavy gastrointestinal bleeding during heart support [56]. Gastrointestinal bleeding occurs in about 25% of all recipients of VAD [57]. Bleeding in the urinary tract occurs in a ratio of 1:6 to gastrointestinal bleeding. Patients with these bleedings must be treated up to three times longer in hospital. Treatment is difficult and measures that are successful in gastrointestinal bleeding sometimes have no effect on bleeding in the urinary tract [58]. Cerebral haemorrhage is severe but rare [59]. Von-Willebrand-syndrome (VWS) is widespread in the use of CF-VAD [60, 61]. In VWS there is a deficiency of the Willebrand factor, a factor VIII carrier protein that causes blood clotting, resulting in a reduced accumulation of thrombocytes on injured tissue, prolonging the duration of bleeding. This disease is hereditary and cannot be acquired. When CF-VAD is used, the VWS occurs relatively frequently, which initially could not be explained. The syndrome disappears immediately after the end of the cardiac support [62]. Therefore, it is called acquired VWS. Studies have shown that the Willebrand factor in blood pumps is destroyed by shear stress. The protein is not destroyed by mechanical stress alone, but also by the enzyme ADAMTS-13, which breaks the protein down into further fragments, but only under the influence of shear stresses. In pulsatile pumps with only low shear stress (< 20 Pa) the VWS has rarely been observed. Only with continuously pumping rotary pumps does the VWS occur more frequently. Currently, the critical limit is assumed to be 25 Pa. VWS is believed to be one of the major causes of bleeding when using CF-VAD [63–65]. The term haemolysis is used to describe the dissolution or degradation of erythrocytes. In blood pumps haemolysis is caused by mechanical stress on the erythrocytes. Their cell walls are damaged by shear stresses in the blood, caused by increased wall friction due to the flow of blood after being pumped. The damage depends both on the level of shear stress and the duration of exposure to the erythrocytes. Haemolysis also occurs much less frequently in pulsatile VAD than in continuous flow VAD [66].

### Thrombus formation

Patients with VAD or TAH generally receive anticoagulant medication. A lower dose can lead to thrombus formation, but if the dose is too high, bleeding will increase. It becomes fatal when thrombi form in the heart vessels or in the blood vessels of the brain [67–69]. Dang et al. [70] provide an overview of the current treatment methods for thromboses caused by the implantation of VAD. Foreign objects in the body, such as blood pumps, can cause blood clotting when blood clotting factor XII comes into contact with the foreign surfaces. Another triggering factor is the activation of thrombocytes by shear stress. The critical shear stress for this is lower than that which can lead to haemolysis. Critical shear stresses are given as 10–15 Pa [71], for short-term loads below 0.01 s 57–108 Pa [72]. If increased haemolysis occurs, the risk of thrombus formation increases [73]. The occurrence of thrombus formation has decreased since the use of CF-VAD to 2–13% of all patients, but it occurs in 18% of pediatric patients with extracorporal pulsatile devices [70]. Pump exchange due to thrombosis is necessary for about 10% of patients who received a CF-VAD [74] and in up to 100% of pediatric patients [75]. One strategy to prevent this is to coat the surfaces that come into contact with blood with heparin or other anticoagulants which should retain their effect permanently. Berlin Heart pumps have been doing this for some time now [14, 15], but other studies show no long-time durability of heparin coatings [76]. The alternative is to coat the surfaces with endothelial cells. This was already passively attempted in the first blood pumps by coating the surfaces with plastic fibre to promote the accumulation of blood cells. In the latest experiments, endothelial cells are colonized on sintered titanium surfaces, which must be done shortly before the blood pump is implanted [77] but this is far away from clinical use. The survival rate of patients with thrombosis is lower than that of patients without it [78].

### Infections

According to the International Society for Heart and Lung Transplantation (ISHLT), a VAD-induced infection is defined as an infection caused by the presence of VAD. This includes also many types of infections not directly associated with the VAD, that can be detected in the bloodstream, respiratory system or urinary tract. They can be classified by their location, such as pump casing, power supply cable or inflow and outflow cannula [79]. The most common type in CF-VAD is driveline infections. A biofilm forms at the site of skin penetration, which may contain the typical pathogens such as *staphylococcus *sp. or *pseudomonas *sp. In most cases, these infections can be successfully treated and do not require VAD replacement [80]. No connection between driveline infections and the occurrence of thrombus formation or strokes was found [81]. In SynCardia TAH, driveline infections account for less than 10% of all infections [82]. This may be due to the fact that TAH remains in the patient much shorter than VAD. This is because driveline infections occur in CF-VAD on average 190 days after the insertion of the systems, while the average duration of use of a TAH is only 100 days [83]. In many cases, the causes of infection after implantation of a VAD or TAH cannot be determined. The pathogens may have already entered the body during the operation so that the blood pumps are not the cause. While infections were still the main cause of death in pulsatile heart support systems [83], today they essentially mean that the patient has to stay in the hospital longer. Over the years, the rate of infections did not change much. In Europe the rate till 1997 with only pulsatile pumps was 30%, with widely used continuous flow pumps from 2011 till 2016 it was 31% [84, 85].

### Outcome

The first large comparative study (REMATCH study—Randomized Evaluation of Mechanical Assistance for the Treatment of Congestive Heart Failure) on survival rates was conducted from 1998 to 2001. A study was done on the survival rate of patients with congestive heart failure comparing a group who received medication and a group who received P-VAD. This study demonstrated that patients with P-VAD had a 48% lower mortality risk in the time studied and a 27% lower mortality rate after 1 year [83]. This investigation was the trigger for the rapidly increasing use of VAD. A corresponding study was conducted as a comparison between continuously promoting and pulsatile VAD ending 2009, over 2 years of each patient’s life. After 1 year the survival rate with continuously pumping pumps was 68%, with pulsatile pumps 58%, after 2 years 55% and 24%, respectively [86]. The pulsatile systems were thus pushed out of the market. Today there are only a few extracorporeal systems and no implantable systems left, except for TAH. In the 6th INTERMACS annual report of 2014, between 2006 and 2013, patients with continuous pumping pumps had a survival rate of 80% after 1 year, 70% after 2 years, 60% after 3 years and 50% after 4 years. In patients with pulsatile pumps, survival rates were 65, 45, 40 and 35%. TAH had a survival rate of 60% after 1 year [87]. The 30th Adult Heart Transplant Report for 2013 shows a significant jump in the number of heart transplants after VAD or TAH between 2008 and 2009. By 2008, 20% of all patients were treated with VAD or TAH prior to heart transplantation. Since 2009, the number has risen to over 30%. The survival rate of these patients was lower until 2011, mainly in patients who needed a bi-VAD. Up to 3 years after heart transplantation the survival rate of patients with CF-VAD is higher, after 5 years the survival rate of patients with P-VAD is higher. After 1 year: CF-VAD 88%/P-VAD 87%, after 2 years 85%/83%, after 3 years 82%/81%, after 4 years 78%/78%, after 5 years 72%/75%, after 6 years 68%/72%. The overall survival rate after 6 years is still 70% [88]. In Japan, the average waiting time for heart transplantation is 877 days, much longer than in other countries where the average is 50 days. Therefore, 90% of patients receive VAD beforehand. Nevertheless, survival rates in Japan are much higher. At the heart centre in Osaka, 95% of the transplanted patients survived after 12 years. For Japan as a whole, the survival rate after 12 years is 64%, compared to 45% worldwide [89]. For infants and children currently only the Berlin Heart EXCOR Pediatric is an approved VAD. Between 2004 and 2014, 2885 donor hearts were transplanted in children under the age of 18 in the USA. 358 of them received a Berlin Heart EXCOR Pediatric as bridge to transplantation. 225 children received an LVAD, nine a RVAD and 123 a Bi-VAD. 30 days, 1 year and 5 years after transplantation, no differences in survival between supported and unsupported patients were observed. After 5 years the survival rate was still over 70% [90]. In England, 84% of all children with P-VAD survived until transplantation or heart recovery. In another study, 75% of children survived 1 year with heart support, 64% achieved transplantation and 6% recovered completely [20].

### Bridge to recovery

In 1995, two patients at the German Heart Centre Berlin (Deutsches Herzzentrum Berlin) underwent an unscheduled removal of their Novacor or HeartMate IP-LVAS. To everyone’s surprise, their hearts had recovered so much during the period of cardiac support that they continued to beat normally after removal of the P-VAD, so that no further support was necessary. As a result, two other patients, whose hearts also showed strong signs of recovery, underwent a planned removal of their P-VAD. Since then, the term “bridge to recovery” has become established when the heart has recovered sufficiently after the implantation of a VAD so that it can be removed again and the patient lives on as cured [1]. While in the early days of cardiac support, when only pulsatile pumps were used, heart recovery was predicted to occur in up to 15% of patients, the overall rate between 2006 and 2015 was 1.3%. No significant difference was observed between pulsatile and continuous flow pumps. The rate for the use of the Berlin Heart INCOR is at least 5% [91–93]. To date, a reliable prediction as to whether the heart will fully recover is not possible [90]. Whether the natural heart has recovered sufficiently for VAD explantation is not easy to determine. With pulsatile pumps, these are briefly switched off and the reaction of the heart is observed. With continuous flow pumps, switching off does not lead to such clear results because the blood can flow through the pump from the aorta back into the ventricle. A prolonged shutdown of continuously pumping pumps is, therefore, dangerous [91, 94, 95]. The recovery of the heart to such an extent that the patient no longer needs VAD and, of course, heart transplantation, is, therefore, a goal that many researchers are increasingly striving for in the future in the treatment of severe heart diseases.

## Conclusion

Pulsatile VAD was the first to save the lives of many people with diseased hearts. Although there is no implantable pulsatile VAD on the market anymore, the corresponding extracorporeal systems, which are still used in continuously improved form, have some serious advantages. The use of pulsatile VAD reduces bleeding. Von Willebrand’s syndrome and haemolysis almost never occur because the shear stress on the blood in these blood pumps is very low. Because the extracorporeal pumps are constantly monitored visually, a malfunction can be detected and corrected immediately. If necessary, the pumps can be easily replaced. If the VAD fails, the pump does not act as a short-circuit because the artificial heart valves prevent backflow. In addition, the systems are cheaper because the drive unit is reused. Only pulsatile volume displacement pumps are able to generate high blood pressure. That is why they are still the only systems suitable for infants and small children. The main disadvantages of the extracorporeal pulsatile pumps are that the patients have to stay in hospital all the time. For that, the quality of life is poor compared to implantable pumps. The costs for the hospital stay may so be higher than the benefit of the lower costs of the system. For long-time use as bridge to transplantation or destination therapy today only implantable rotary blood pumps are used. Extracorporeal pulsatile pumps are now used mainly in infants and children or sometimes as a bridge to recovery or biventricular support as bridge to transplantation. For short time use the pulsatile pumps were substituted by extracorporeal rotary pumps like Tandem Heart or CentriMag or by catheter-mounted pumps of the Impella-family.

## References

[1] Müller J, Wallukat G, Wenig YG, Hetzer R, Henning E, Loebe M (1997). Weaning from mechanical support after complete recovery in patients with idiopathic dilated cardiomyopathy. Mechanical circulatory support: in children, towards myocardial recovery, permanent.

[2] Valika AA, Cotts W (2013). A review of long-term mechanical circulatory support as destination therapy: evolving paradigms for treatment of advanced heart failure. ISRN Transplantation..

[3] DeBakey ME (1971). Left ventricular bypass pump for cardiac assistance. Am J Cardio.

[4] Liotta D (2002). Early clinical application of assisted circulation. Tex Heart Inst J.

[5] DeBakey ME (2005). Development of mechanical heart devices. Ann Thorac Surg.

[6] Wolner E, Deutsch M, Losert U (1978). Clinical application of the ellipsoid left heart asisst device. Artif Organs.

[7] Wieselthaler GM, Schima H, Zimpfer D (2008). Forty years of development, experimental evaluation and clinical application of mechanical circulatory support at the Medical University of Vienna. Wien Klin Wochenschr.

[8] Moritz A, Rokitansky A, Schima H (1991). Succesful bridge transplantation with the Vienna artificial heart. Wien Klin Wochenschr.

[9] Pennington DG, McBride LR, Swartz MT (1989). Use of the Pierce-Donachy ventricular assist device in patients with cardiogenic shock after cardiac operations. Ann Thorac Surg.

[10] Farrar DJ, Hill JD (1993). Univentricular and Biventricular Thoratec VAD Support as a Bridge to Transplantation. Ann Thorac Surg.

[11] Wu EL, Stevens MC, Pauls JP, Gregory SD, Stevens MC, Fraser JF (2018). First-generation ventricular assist devices. Mechanical Circulatory and Respiratory Support.

[12] Thuaudet S (2000). The Medos ventricular assist device system. Perfusion.

[13] Reiß N, El-Banayosy A, Arusoglu L (2001). Mechanische Kreislaufunterstützung Mit Dem Hia-Medos-System—Erfahrungen Mit Drei Verschiedenen Ventrikelgrössen. Biomed Techn.

[14] Werkkala K, Jokinen JJ, Soininen L (2016). Clinical durability of the CARMEDA bioactive surface in EXCOR ventricular assist device pumps. ASAIO.

[15] Hetzer R, Kaufmann F, Delmo Walter EM, Kramme R (2017). Mechanische Herzunterstützungssysteme. Medizintechnik.

[16] Hennig E, Zartnak F, Schiessler A, Hetzer R (1990). Das Berliner Herzunterstützungssystem.

[17] Kaufmann F, Hennig E, Loebe M, Hetzer R (1996). Das Berlin heart system.

[18] Drews T, Loebe M, Hennig E (2000). The ‘Berlin Heart’ assist device. Perfusion.

[19] Rukosujew A, Hoffmeier A, Tjan TDT, Boeken U, Assmann A, Born F (2017). Parakorporale Systeme einschließlich Implantationstechniken: Berlin Heart EXCOR^®^ VAD. Mechanische Herz-Kreislauf-Unterstützung.

[20] Chopski SG, Moskowitz WB, Stevens RM (2017). Review article—mechanical circulatory support devices for pediatric patients with congenital heart disease. Artif Organs.

[21] Nishida M (2017). Artificial hearts—recent progress: republication of the article published in the Japanese Journal of Artificial Organs. J Artif Organs.

[22] Berlin Heart. Press release: Longest support time with the EXCOR Pediatric ventricular assisst device, August 2012.

[23] Berlin Heart. Press release: Berlin Heart unterstützt nun auch Kinder in Südkorea und Kroatien, January 2018.

[24] Samuels LE, Holmes EC, Ganwood P (2005). Initial experience with the Abiomed AB5000 ventricular assist device system. Ann Thorac Surg.

[25] Zhang L, Kapetanakis EI, Cooke RH (2007). Bi-Ventricular circulatory support with the Abiomed AB5000 system in a patient with idiopathic refractory ventricular fibrillation. Ann Thorac Surg.

[26] Wassenberg PAJ (2000). The Abiomed BVS 5000 biventricular support system. Perfusion.

[27] Dekkers RJ, FitzGerald DJ, Couper GS (2001). Five-year clinical experience with Abiomed BVS 5000 as a ventricular assist device for cardiac failure. Perfusion.

[28] Kustosz R, Nawrat Z, Drzazga M, Akutsu T, Koyanagi H (1996). Early results of experimental clinical usage of polish ventricle assist device—POLVAD-II. Heart replacement—artificial heart 5.

[29] Malota Z, Sadowski W, Krzyskow M (2016). The application of bileaflet mechanical heart valves in the polish ventricular assist device: physical and numerical study and first clinical usage. Artif Organs.

[30] Sato N, Mohri H, Sezai Y (1990). Multi-institutional evaluation of the Tokyo university ventricular assist system. ASAIO Transactions.

[31] Nishimura T (2014). Current status of extracorporeal ventricular assist devices in Japan. J Artif Organs.

[32] Saito S, Matsumiya G, Sakaguchi T (2009). Fifteen-year experience with Toyobo paracorporeal left ventricular assist system. J Artif Organs.

[33] Takano H, Nakatani T (1996). Ventricular assist systems: experience in Japan with Toyobo pump and zeon pump. Ann Thorac Surg.

[34] Xuejun X, Ruixin F, Anheng C (2002). The clinical trial of pneumatic pump (Luo-Ye pump) as left ventricular assist device. South China J Cardiol.

[35] Gu K, Chang Y, Gao B (2014). Development of ventricular assist devices in China: present status, opportunities and challenges. Eur J Cardio-Thorac Surg.

[36] DeBakey ME, Liotta D, Hall CW. Left-heart bypass using an implantable blood pump. In: Mechanical devices to assist the failing heart. Proceedings of a Conference Sponsored by The Committee on Trauma, Division of Medical Sciences, National Academy of Sciences-National Research Council. Washington; 1966. p. 223–239.

[37] Norman JC (1976). An intracorporal (abdominal) left ventricular assist device [Alvad], XXX: clinical readiness and initial trials in man. Cardiovasc Dis Bull Tex Heart Inst.

[38] Norman JC, Duncan MD, Frazier OH (1981). Intracorporeal (Abdominal) left ventricular assist devices or partial artificial hearts: a five-year clinical experience. Arch Surg.

[39] Wheeldon DR, Jansen PGM, Portner PM (2000). The Novacor electrical implantable left ventricular assist system. Perfusion.

[40] Wheeldon DR, LaForge DH, Lee J (2002). Novacor left ventricular assist system long-term performance: comparison of clinical experience with demonstrated in vitro reliability. ASAIO J.

[41] Poirier VL (1997). The heartmate left ventricular assist system: worldwide clinical results. Eur J Cardio-Thorac Surg.

[42] Frazier OH, Myers TJ, Radovančević B (1998). The HeartMate^®^ left ventricular assist system. Overview end 12-Year experience. Tex Heart Inst J.

[43] Frazier OH (1994). First use of an untethered, vented electric left ventricular assist device for long-term support. Circulation.

[44] Frazier OH, Rose EA, Oz MC (2001). Multicenter clinical evaluation of the HeartMate vented electric left ventricular assist system in patients awaiting heart transplantation. J Thorac Cardiov Surg.

[45] Samuels LE, Holmes EC, Hagan K, et al. The thoratec implantable ventricular assist device(ivad): initial clinical experience. Heart Surg Forum. 2006; 9(4):E690-2. 10.1532/HSF98.20061028 16757424

[46] Slaughter MS, Tsui SS, El-Banayosy A (2007). Results of a multicenter clinical trial with the Thoratec Implantable Ventricular Assist Device. J Thorac Cardiov Surg.

[47] Mehta SM, Pae WE, Rosenberg G (2001). The LionHeart LVD-2000: a completely implanted left ventricular assist device for chronic circulatory support. Ann Thorac Surg.

[48] Mehta SM, Silber D, Boehmer JP (2006). Report of the first US patient successfully supported long term with the LionHeart completely implantable left ventricular assist device system. ASAIO J.

[49] Pagani FD, Miller LW, Russell SD (2009). Extended mechanical circulatory support with a continuous-flow rotary left ventricular assist device. J Am Coll Card.

[50] Holman WL, Naftel DC, Eckert CE (2013). Durability of left ventricular assist devices: Interagency Registry for Mechanically Assisted Circulatory Support (INTERMACS) 2006 to 2011. J Thorac Cardiov Surg.

[51] Giridharan GA, Koenig SC, Soucy KG (2015). Hemodynamic changes and retrogate flow in lvad failure. ASAIO J.

[52] Kormos RL, McCall M, Althouse A (2017). Left ventricular assist device malfunctions: it is more than just the pump. Circulation.

[53] Throckmorton AL, Patel-Raman SN, Fox CS (2016). Beyond the VAD: human factors engineering for mechanically assisted circulation in the 21st century. Artif Organs.

[54] Burrell AJC, Salamonsen RF, Murphy DA, Gregory SD, Stevens MC, Fraser JF (2018). Complications of mechanical circulatory and respiratory support. Mechanical circulatory and respiratory support.

[55] Patel SR, Oh KT, Ogriki T (2018). Cessation of continuous flow left ventricular assist device-related gastrointestinal bleeding after heart transplantation. ASAIO J.

[56] Holley CT, Harvey L, Roy SS (2015). Gastrointestinal bleeding during continuous-flow left ventricular assist device support is associated with lower rates of cardiac transplantation. ASAIO J.

[57] Truss WD, Weber F, Pamboukian SV (2016). Early implementation of video capsule enteroscopy in patients with left ventricular assist device and obscure gastrointestinal bleeding. ASAIO J.

[58] Son AY, Zhao L, Reyentovich A (2016). Intractable hematuria after left ventricular assist device implantation: can lessons learned from gastrointestinal bleeding be applied?. ASAIO J.

[59] Kitamura T, Torii S, Oka N (2015). Seventeen-month-long paracorporeal biventricular mechanical support as a bridge to transplantation for severe dilated cardiomyopathy. J Artif Organs.

[60] Uriel N, Pak SW, Jorde UP (2010). Acquired von Willebrand syndrome after continuous-flow mechanical device support contributes to a high prevalence of bleeding during long-term support and at the time of transplantation. J Am Coll Cardiol.

[61] Meyer AL, Malehsa D, Budde U (2014). Acquired von Willebrand syndrome in patients with a centrifugal or axial continuous flow left ventricular assist device. Heart Failure J Am Coll Cardiol.

[62] Davis ME, Haglund NA, Tricarico NM (2015). Immediate recovery of acquired von willebrand syndrome after left ventricular assist device explantation: implications for heart transplantation. ASAIO J.

[63] Bartoli CR, Dassanayaka S, Brittian KR (2014). Insights into the mechanism(s) of von Willebrand factor degradation during mechanical circulatory support. J Thorac Cardiov Surg.

[64] Bartoli CR, Restle DJ, Zhang DM (2015). Pathologic von Willebrand factor degradation with a left ventricular assist device occurs via two distinct mechanisms: mechanical demolition and enzymatic cleavage. J Thorac Cardiov Surg.

[65] Rosenberg G, Siedlecki CA, Jhun C-S (2018). Acquired von willebrand syndrome and blood pump design. Artif Organs.

[66] Takami Y, Nakazawa T, Makinouchi K (1997). Hemolytic effect of surface roughness of an impeller in a centrifugal blood pump. Artif Organs.

[67] Schweiger M, Hübler M, Albisetti M (2015). Heparin anticoagulation monitoring in patients supported by ventricular assist devices. ASAIO J.

[68] Jennings DL, Horn ET, Lyster H (2016). Assessing anticoagulation practice patterns in patients on durable mechanical circulatory support devices: an international survey. ASAIO J.

[69] Kantorovich A, Fink JM, Militello MA (2016). Comparison of anticoagulation strategies after left ventricular assist device implantation. ASAIO J.

[70] Dang G, Epperla N, Muppidi V (2016). Medical management of pump-related thrombosis in patients with continuous-flow left ventricular assist devices: a systematic review and meta-analysis. ASAIO J.

[71] Sutera SP (1977). Flow-induced trauma to blood cells. Circ Res.

[72] Wurzinger LJ, Opitz R, Blasberg P (1985). Platelet and coagulation parameters following millisecond exposure to laminal schear stress. Thromb Haemost.

[73] Boehme AK, Pamboukian SV, George JF (2017). Anticoagulation control in patients with ventricular assist devices. ASAIO J.

[74] Kirklin JK, Naftel DC, Kormos RL (2014). Interagency Registry for Mechanical Assisted Circulatory Support (INTERMACS) analysis of pump thrombosis in the HeartMate II left ventricular assist device. J Heart Lung Transplant.

[75] Maeda K, Almond C, Hollander SA (2018). Characteristics of deposits and pump exchange in the Berlin Heart EXCOR ventricular assist device: Experience with 67 cases. Pediatr Transplantation.

[76] Engelhardt S (2007). In-vivo Langzeittestung antithrombogener Beschichtungen von mechanischen Kreislaufunterstützungssystemen im Schweinemodell.

[77] Noviani M, Jamiolkowski RM, Grenet JE (2016). Point-of-Care rapid-seeding ventricular assist device with blood-derived endothelial cells to create a living antithrombotic coating. ASAIO J.

[78] Upshaw JN, Kiernan MS, Morine KJ (2016). Incidence, management, and outcome of suspected continuous-flow left ventricular assist device thrombosis. ASAIO J.

[79] Nienaber J, Wilhelm MP, Sohail MR (2013). Current concepts in the diagnosis and management of left ventricular assist device infections. Exp Rev Anti-infective Therapy.

[80] O’Horo JC, Abu Saleh OM, Stulak JM (2018). Left ventricular assist device infections: a systematic review. ASAIO J.

[81] Fried J, Cagliostro B, Levin A (2015). Driveline infection is not associated with increased risk of thrombotic events in CF-LVAD patients. J Heart Lung Transpl.

[82] Hidalgo LF, Shah KB, Cooke RH (2017). Infections in patients with a total artificial heart are common but rarely fatal. ASAIO J.

[83] Rose EA, Gelijns AC, Moskowitz AJ (2001). Long-term use of a left ventricular assist device for end-stage heart failure. New Engl J Med.

[84] Quaini E, Pavie A, Chieco S (1997). The concerted acton “Heart” European registry on clinical application of mechanical circulatory support systems: bridge to transplant. Eur J Cardio-thorac Surg.

[85] De By TMMH, Mohasci P, Gahl B (2018). The European Registry for Patients with Mechanical Circulatory Support (EUROMACS) of the European Association for Cardio-Thoracic Surgery (EACTS): second report. Eur J Cardio-Thorac Surg.

[86] Slaughter MS, Rogers JG, Milano CA (2009). Advanced heart failure treated with continuous-flow left ventricular assist device. New Engl J Med.

[87] Kirklin JK, Naftel DC, Pagani FD (2014). Sixth INTERMACS annual report: a 10,000-patient database. J Heart Lung Transpl.

[88] Lund LH, Edwards LB, Kucheryavaya AY (2013). The registry of the international society for heart and lung transplantation: thirtieth official adult heart transplant report-2013; focus theme: age. J Heart Lung Transpl.

[89] Kitamura S (2012). Heart transplantation in Japan: a critical appraisal for the results and futur prospects. Gen Thorac Cardiovas Surg.

[90] Bryant R, Zafar F, Castleberry C (2017). Transplant survival after Berlin heart EXCOR support. ASAIO J.

[91] Dandel M, Potapov E, Krabatsch T (2012). Myokarderholung unter mechanischer Ventrikelentlastung und Entwöhnung vom ventrikulären Unterstützungssytem. Zeitschr Herz- Thorax- Gefäßchir.

[92] Phan K, Huo YR, Zhao DF (2016). Ventricular recovery and pump explantation in patients supported by left ventricular assist devices: a systematic review. ASAIO J.

[93] Wever-Pinzon O, Drakos SG, McKellar SH (2016). Cardiac recovery during long-term left ventricular assist device support. J Am Coll Cardiol.

[94] Saito S, Toda K, Miyagawa S (2015). Hemodynamic changes during left ventricular assist device-off test correlate with the degree of cardiac fibrosis and predict the outcome after device explantation. J Artif Organs.

[95] Segan LA, Nanayakkara SS, Leet AS (2017). Exercise hemodynamics as a predictor of myocardial recovery in LVAD patients. ASAIO J.

